# Cross-Border Quest: The Reality and Legality of Transplant Tourism

**DOI:** 10.1155/2012/391936

**Published:** 2012-05-10

**Authors:** Frederike Ambagtsheer, Damián Zaitch, René van Swaaningen, Wilma Duijst, Willij Zuidema, Willem Weimar

**Affiliations:** ^1^Department of Internal Medicine, Section Transplantation, Erasmus Medical Centre, University Hospital, 's-Gravendijkwal 230, 3015 CE Rotterdam, The Netherlands; ^2^Willem Pompe Institute for Criminal Law and Criminology, Utrecht University, Utrecht, The Netherlands; ^3^Criminology Department, Erasmus School of Law, Erasmus University Rotterdam, The Netherlands; ^4^Department of Public Health, Radboud University Nijmegen, The Netherlands

## Abstract

*Background.* Transplant tourism is a phenomenon where patients travel abroad to purchase organs for transplants. This paper presents the results of a fieldwork study by describing the experiences of Dutch transplant professionals confronted by patients who allegedly purchased kidney transplants abroad. Second, it addresses the legal definition and prohibition of transplant tourism under national and international law. The final part addresses the legal implications of transplant tourism for patients and physicians. *Methods.* The study involved seventeen interviews among transplant physicians, transplant coordinators and policy-experts and a review of national and international legislation that prohibit transplant tourism. *Results.* All Dutch transplant centers are confronted with patients who undergo transplants abroad. The estimated total number is four per year. Transplant tourism is not explicitly defined under national and international law. While the purchase of organs is almost universally prohibited, transplant tourism is hardly punishable because national laws generally do not apply to crimes committed abroad. Moreover, the purchase of organs (abroad) is almost impossible to prove. *Conclusions.* Transplant tourism is a legally complex phenomenon that warrants closer research and dialogue. The legal rights and obligations of patients and physicians confronted with transplant tourism should be clarified.

## 1. Introduction

In 2000 the United Nations (UN) General Assembly adopted the UN Convention against Transnational Organized Crime (UNTOC) as a response to the expansion of global crime. This convention and its protocols aim to promote cooperation to prevent and combat transnational organized crime more effectively [1]. One of its protocols is the Protocol to Prevent, Suppress and Punish Trafficking in Persons, especially women and children [2]. With this protocol, the UN opted for a broad definition of trafficking in human beings (THB), recognizing that in addition to sexual exploitation, an international instrument was needed to address all forms of THB. The definition of THB was widened to include the forced removal of organs [2].

Almost all states have signed and ratified the UNTOC. Ratification involved the obligation of member states to bring in line the new THB definition with their national laws. The wider THB definition prompted the National Reporter on Trafficking in Human Beings to study organ trade and trafficking in the Netherlands. The Reporter advises and reports to the government on THB. The aim of the study was to verify whether the Netherlands formed part of the global organ trade and trafficking problem. The study consisted of two parts. The first, a review of media sources, police reports, and academic (legal and medical) literature, suggested that there were no indications of organ trafficking or trade [3]. The second part (reported herein) was an exploratory field study in which we interviewed transplant professionals about their experience with patients who had bought kidneys for transplants abroad (here defined as transplant tourism).

The structure and aim of the present study is threefold. First, it presents the results of the fieldwork study by describing the experiences of transplant professionals with transplant tourism. Second, it addresses the prohibition of transplant tourism. The final part addresses the legal implications of buying organs for transplants abroad for patients and physicians.

To date, no qualitative studies that deal with legal aspects of transplant tourism have been published

## 2. Subjects and Methods

### 2.1. Field Study

The field study involved in-depth interviews with transplant physicians, transplant coordinators, and organ donation experts. We opted for a qualitative study because we expected that face-to-face interviews would produce more reliable information about this sensitive topic than quantitative research methods. Because kidneys are the most commonly traded organ, we focused on kidneys only. The research involved seventeen in-depth interviews among nine transplant physicians (nephrologists and surgeons), four transplant coordinators, and four policy experts. One expert was a former state secretary of health, the others worked for the Health Council, the Transplant Foundation, and the Institute for Health Promotion.

Interviews with transplant physicians and coordinators took place at all (seven) national transplant centres. Interview data was handled and analyzed anonymously. A topic list with open questions was used. This is presented in Table 1.

### 2.2. International and National Prohibition of Transplant Tourism

For the legal research, we first made a list of the relevant international documents that explicitly define and/or prohibit transplant tourism. We used legal data sources including Heinonline, Westlaw International, and International Law Reports Online, using the English terms “transplant tourism” and “organ tourism.” By international documents, we mean those documents that have been developed by international organisations such as conventions, resolutions, and declarations.

On the international level transplant tourism is poorly defined and addressed. We found three organisations that address transplant tourism: the World Health Organisation (WHO), the Transplantation Society (TTS), and International Society of Nephrology (ISN). The WHO mentions transplant tourism in World Health Assembly (WHA) Resolution 57.18. The WHA Resolution calls upon states to protect vulnerable groups from transplant tourism but does not prohibit the purchase of organs by patients abroad [4].

The second document that condemns transplant tourism is the Declaration of Istanbul on Organ Trafficking and Transplant Tourism (DOI), established in 2008 during a summit organised by the TTS and the ISN. The DOI is the first document, drawn up by transplant professionals, that defines and condemns transplant tourism [5]. Whereas the DOI is intended to influence transplant professionals and transplantation societies, the WHO intends to influence governments [6]. Both act in concert to address transplant tourism. Even though the DOI is not legally binding, it has proven to have significant influence. Over 100 transplant organizations endorse its principles. Countries including Israel, China, and the Philippines have passed regulations to curb transplant tourism.

The DOI distinguishes between *travel for transplantation* and *transplant tourism*. Whereas it considers travel for transplantation as a legitimate phenomenon, it states that transplant tourism “should be prohibited” when it involves trafficking and/or commercialism or if the national resources to provide organs to own nationals are undermined [5]. It can thus arguably be suggested that the DOI perceives transplant tourism as a crime. The DOI and WHA Resolution's full definition and condemnation of transplant tourism is stated in Table 2 Aside from the DOI and WHA Resolution, there are no international regulations that condemn or prohibit transplant tourism.

Under the EU-funded project on Living Organ Donation in Europe (http://www.eulod.eu/), we also searched for national laws that explicitly define and prohibit transplant tourism. Research into national legislation against transplant tourism was conducted in Moldova, Romania, Hungary, Germany, the Netherlands, and Serbia in the languages of these countries. Through contacts with our network of lawyers and searches on the web, we were able to identify national legislation. National laws generally distinguish between THB for organ removal (organ trafficking) and the prohibition of financial gain (organ trade or commercialism), yet none of the laws that we found explicitly defines or prohibits transplant tourism [7].

National laws against organ trade and trafficking—in principle—have a *territorial scope*. This means that these provisions apply to crimes committed within a state's territory only. If a patient is found to have committed transplant tourism, that is, the purchase of an organ for transplant *abroad*, the national prohibition of organ purchase and/or organ trafficking should be applied and interpreted in conjunction with the provisions on extraterritorial jurisdiction. Extraterritorial jurisdiction refers to the authority of the state to prosecute its inhabitants for crimes committed abroad. Extraterritorial jurisdiction is bound by strict conditions. Its scope varies per state and varies depending on the severity of the crime.

## 3. Results of Field Study

### 3.1. Scale and Nature

First of all, no indications were found in this study of patients trafficking people abroad for the purpose of their organs.

All transplant physicians and three transplant coordinators were confronted with patients who had expressed a desire to obtain organs for transplantation abroad. They also knew or had heard of patients who had succeeded in travelling abroad for kidney transplantation. The policy experts did not have any information or knowledge about patients going abroad. Most respondents in the study presumed that patients had *bought* their transplant kidneys, but they did not have any evidence to validate the presumption that the organs were purchased. Cases of patients who go abroad for kidney transplants are not reported or registered anywhere. The respondents referred to the number of patients going abroad for kidney transplants as “incidental.” The number of reported cases per centre ranges from two patients a year to less than five over three decades. Nationally, the estimated total number is four per year. Many respondents indicated that the number is small, because the majority of patients find it too risky to undertake the endeavour to a country where they are unfamiliar with the health system.

Most cases became known to doctors and coordinators after the transplant abroad had taken place and the patient returned to the transplant centre with complications. On some occasions, cases became apparent in the pretransplant stage because patients told their physicians that they had found information on the internet and were exploring the possibility of undergoing a transplant abroad.

### 3.2. Destinations

Physicians and coordinators experienced patients who had travelled to China, India, Iran, Pakistan, Iraq, United States, Colombia, and Afghanistan. Most patients who underwent a kidney transplant abroad had an affinity with the country or region they went to, either because this was the country of origin or because they had worked or lived in that country. Some patients were refugees but physicians also referred to patients from “the upper class” that travelled abroad for transplants. Almost all cases involved patients travelling abroad independently of one another. One transplant center knew a group of about five patients who travelled to India shortly after one another. These patients received dialysis treatments in the same local hospital. It was alleged by the physician that they helped each other find transplant opportunities or at least had given each other ideas about going abroad.

Not all patients travelled to their countries of origin to obtain transplant surgery. For example, refugees from Libya and Morocco travelled to China. One man was investigating transplant possibilities in Colombia because he had lived there before and was acquainted with hospitals there. Another man had undergone a transplant in India ten years ago while he was living and working for a multinational corporation in Singapore.

### 3.3. How Physicians Cope with Transplant Tourism

We found that transplant physicians are affected by and deal with transplant tourism in different ways, based on personal, individual considerations.

All physicians and transplant coordinators expressed ethical, medical, and legal concerns about patients travelling abroad for transplants. Ethical concerns involved the likelihood that the organs were procured from impoverished, exploited donors. Nephrologists highlighted that although they were uncertain, they assumed that patients had bought organs presumably through unregulated, black market transactions.

Medical complications are a second reason why the respondents condemn and advice against transplant tourism, namely, the medical risks of getting a transplant in countries where black market transactions occur and safety and protection measures are likely to be poor. Experiences with the well-being of patients returning from a transplant abroad vary. While some nephrologists have the impression that medical complications frequently occur, others stressed that transplants performed abroad are *“state-of-the-art.”*

Legal considerations also affect the way physicians deal with potential cases of transplant tourism. Almost all physicians and coordinators pointed out that they condemn transplant tourism because the law prohibits the purchase of organs. They do not support their patients with their search for transplant opportunities abroad. However, physicians emphasized that they will provide medical records if a departing patient requests it, because patients have a legal right to receive them. All physicians further stated that they will always provide medical care for their patients if they return with complications.

Most nephrologists indicated that they face a dilemma, affected by the duty of medical care on the one hand and the prohibition of organ trade on the other. In one instance, legal reasons were a reason for the nephrologist to help his patient of Iranian nationality to undergo a transplant in his home country, “*because it is legal for Iranian nationals to undergo paid transplants there.*”

Some physicians referred to their professional secrecy oath. They said that they do not register cases of alleged transplant tourism because they are legally withheld from bringing out information about patients. “*It's not my responsibility anyway,*” one nephrologist said, when asked if he had asked his patients' about their experiences abroad. Most doctors indicated that patients do not tell them about their plans and leave without informing their physicians. Moreover, they even preferred not to know about their patients" experiences. There seems to be an agreed upon “do not ask, do not tell” policy. Many nephrologists emphasized the existence of a language barrier and the lack of communication with their patients. One nephrologist pinpointed the lack of communication as a convenient excuse.

National rules and guidelines for physicians on how to deal with transplant tourism do not exist. Knowledge on the (il)legality of undergoing transplants abroad is lacking. The following paragraph addresses the possible legal consequences of transplant tourism for patients and physicians.

## 4. Results of Legal Study

### 4.1. The Legal Implications for Patients

The purchase of organs is a prohibited act in almost every single country. Were legal consequences to arise for the patient, they will be regulated by the criminal law of the state where the organ was purchased. Yet, these laws are not equipped to deal with transplant tourism. They apply to acts committed *within* the territory of the state, not outside it. In principle, if a patient buys an organ on the territory of another state (*the destination state*), he or she is criminally liable and can be persecuted under the law of the destination state and not of the *(resident)* state. The main legal implication of transplant tourism, therefore, is that when the patient leaves a country after buying an organ that is unnoticed or ignored by local enforcement institutions, the legal consequences cease to exist.

Consequently, whereas the purchase of organs is illegal, the purchase of organs will not (always) be *punishable. *Countries only have the authority to persecute their nationals for crimes committed abroad if certain conditions are fulfilled. The Netherlands for instance may only prosecute its patients for organs purchased abroad if the patient has the *Dutch nationality* and if the destination country *punishes* the same act.

Even if these conditions of extraterritorial jurisdiction are fulfilled, this does not automatically legitimize the state to prosecute its patients. It needs to be *proven* that the organ was bought. The burden of proof is a salient principle of law that is universally applicable in all jurisdictions. The returning patient's possession of an implanted organ, by nature a legal good, does not constitute proof of purchase. Also, a financial transaction paid by the patient for the transplant will also not constitute proof of organ purchase. Under law, it is the (financial) *profit* made as a result of the organ purchase that will need to be proven, not only to establish the illegality of the act, but also the *criminal liability *of the patient.

### 4.2. The Legal Implications for Physicians

If legal consequences for patients who buy organs abroad hardly arise, does this mean that physicians are equally exempted from legal responsibility? Our field study found that in some instances physicians helped their patients undergo an allegedly purchased organ for transplant abroad. Can physicians (in the resident state) be considered potential complicit actors and be held accountable if they provide support to patients for organ transplants abroad that are (or may be) illegally obtained? Alternatively, can physicians be held accountable if they refuse to provide medical care to their patients?

The rights and duties of physicians confronted with transplant tourism will most likely be affected in the pretransplant process if the patient asks the physician for support, such as providing medical records and/or drafting a medical report prior to the patient's departure to obtain an organ from a potentially paid donor. Mediation or facilitation of commercial organ donations by third parties is often prohibited. This means that physicians who support patients or donors with the purchase or sale of organs *could* be held criminally accountable. Such provisions, however, cannot be automatically applied to *cross-border* organ purchase. Furthermore, potential accountability under criminal law of the physician is further diminished by the difficulty to establish whether the patient will or has indeed committed a crime.

In addition to criminal law, health law regulations also affect the legal responsibility of physicians. Rights and obligations of physicians commonly include the duty to provide medical care, the professional secrecy oath, and the privilege of nondisclosure [8]. These rights and obligations are firmly entrenched in health care rules and regulations. In the Netherlands, for example, the forthcoming *Patients' Rights (Care Sector) Act* [9] represents the current development towards further strengthening of patient rights and stricter responsibilities of health care professionals. These responsibilities are clearly directed towards increasing the safety and quality of patient care.

Considering the weight of health care regulations that generally aim towards protecting and strengthening patients' rights, it is unlikely that physicians can be held liable for supporting patients who opt for medical care abroad, even when this care possibly involves support of an allegedly purchased organ. On the contrary, physicians may be in breach of (binding) health regulations and, therefore, could be held accountable if they refuse to give such care to patients. Furthermore, doctors' oath of secrecy and privilege of nondisclosure of patient information exempt them from the legal duty to report alleged crimes committed by their patients.

These findings stand in stark contrast to what is purported by the Declaration of Istanbul [5] and the statement by the Canadian Society of Transplantation and Canadian Society of Nephrology on Organ Trafficking and Transplant Tourism [10]. Both documents were not designed to be legal documents but have strong symbolic influence, especially the DOI. They stimulate physicians to prevent and prohibit transplant tourism yet such encouragement may involve actions by doctors that could be in breach with national health regulations. For example, the statement by the Canadian Society claims that physicians may in some instances refuse to provide medical records to patients and may elect to defer care to another physician. Such actions violate fundamental patients' rights. It is thus unlikely that these guidelines will be followed by physicians, let alone be considered legitimate under many national rules and regulations.

## 5. Discussion

This study found that, like other countries [11–15], there are indications of patients travelling abroad for transplants. We believe that the estimated total number of four per year is only the tip of the iceberg. A “dark number” of transplant tourism cases likely exist as many cases may not be known to physicians and because only a small number of physicians were interviewed.

However, it is almost impossible to prove whether instances of patients travelling abroad for organ transplants constitute “real” cases of transplant tourism. Even in cases where patients were (allegedly) found to have purchased organs abroad, prosecution did not occur [16, 17]. Furthermore, the invisibility and lack of accusations by potential victims renders the initiative of investigations by law enforcers unlikely. The cross-border and complex nature of this act possibly makes it one of the most difficult crimes to prove and prosecute.

This complexity raises challenges for doctors and other health carers confronted with patients who opt for transplants abroad. Transplant tourism shifts the traditional role of doctors as medical carers to “agents,” encouraged to deter and prevent transplant tourism [10]. What would be the appropriate way of action if a health carer is confronted with a patient who considers going abroad for a—presumably—paid organ transplant? The *type of *response warranted depends on ethical, legal, and medical factors. These factors differ for each individual situation. Any guidelines or recommendations developed for doctors on dealing with tourism should thus distinguish between different scenarios. Furthermore, they should take account of patient privacy rights, their right to receive medical care, doctor's professional secrecy oath, the doctor's privilege of nondisclosure, and the doctor's duty of medical care.

First and foremost, when a patient enquires about the possibility to *undergo* a transplant abroad, the doctor should not immediately speculate that this will be an illegal transplant constituting transplant tourism. Without evidence pointing to the contrary, the patient's intention to go abroad should be regarded as a legitimate endeavour. Nevertheless, considering the medical complications that accompany transplants abroad [11–13], health carers should dissuade the patient from going abroad by warning him/her against the medical risks. Contrary to what Gill et al. claim [10], doctors should refrain from informing or “educating” *all *patients about going abroad: this may bring the unintended consequence of putting ideas into the heads of patients who otherwise might not have considered the possibility at all. Such warnings should only be directed towards the individual patient who has expressed an interest or desire to undergo a transplant abroad.

If the patient is adamant on leaving and asks for his medical records and additional medical support, this falls within the duty to provide care. *Refusal *to give the medical record to a departing patient, or any other medical support, even if the physician *is certain* that the patient is going to buy the organ, constitutes medical negligence and thus breaches the physician's duty to give care and the patient's right to receive it. Not providing care to those in need, be they uninsured, imprisoned for atrocious crimes, or planning to buy an organ is likely to be considered a flagrant violation of human rights as laid down in the European Convention of Human Rights and Biomedicine [18]. The European Court of Human Rights has stated that medical care prevails over other interests [19].

Whereas the patient's right to receive medical care remains untouched, it could be claimed that the doctor *may* consider disclosing patient information to the police. This consideration may arise in the situation where the patient outright declares to his doctor that he is going to *buy* an organ for transplant abroad from a trafficked or paid donor.

Generally, the declaration of the patient that he is going to commit a crime falls within the scope of the patient's right to privacy. The professional secrecy oath, derived from the patient's right to privacy, is a right of the patient, of which the doctor merely is keeper [19]. What flows from the right to privacy is the privilege of doctors not to disclose patient information to police authorities. The professional secrecy and privilege of nondisclosure prevail over crime enforcement. Thus, if the physician reports information confidentially entrusted upon him by the patient without patient consent, the doctor can be held criminally liable.

However, from established case law, it is clear that the doctor's privilege of nondisclosure is not absolute. In very exceptional cases, when overriding interests or conflicts of duties are at stake, a duty *may* arise to *breach* the professional secrecy oath when the doctor is confronted with information that, if not reported, will lead to “*direct and severe*” harm to another individual [19].

The question thus arises whether a patient's declaration that he is going to *buy* an organ for transplant abroad from a paid donor constitutes sufficient justification to report the patient to the police. Considering contemporary case law, it is very unlikely that a paid donor provides sufficient justification to breach professional secrecy and report the patient to police authorities. “Direct and severe harm” is generally defined in the context of intended homicide or child abuse. The purchase of an organ from a paid donor will likely not be equated as a similarly severe crime. However, if the organ would be taken by force from a severely exploited (trafficked) donor, or a murdered donor, this is likely to be accepted as sufficient justification to report the patient to police. Yet, considering that the doctor must clearly motivate breach of professional secrecy, the physician would need to require clear evidence, such as a patient declaration or confession that the donor is going to be directly and severely harmed. In the absence of such information, a breach of the professional secrecy oath is likely to be considered illegitimate.

The foregoing focuses on pretransplant scenarios, yet these considerations are equally relevant for posttransplant situations. All patients returning from (presumed illegal) transplants abroad are entitled to medical care. Only in the case of clear evidence of direct and severe harm to the *trafficked *donor may a doctor *consider *reporting the patient to the police.

In conclusion, legal implications for patients and doctors will differ in each individual case. Doctors will need to weigh the information known to them before deciding to treat presumed “transplant tourists” differently from other patients. Providing suboptimal care to patients should not be based on speculations or presumptions that the transplant abroad was illegal.

Rather than relying on strict measures aimed to prevent and punish transplant tourism, we believe it is more effective to focus on a bottom-up strategy that tackles the root cause of the problem, namely, the demand for organ transplantation. Such initiatives should take account of more sensitive, cultural issues that play a role in everyday patient-doctor interactions. Considering that ethnic minority groups appear to be more likely to travel overseas for organ transplants [14], we advise transplant professionals to take on a more active role in raising awareness about alternatives to transplants abroad. Research has found that these populations are underrepresented in living donation programmes. Involving these groups in alternative living donation programmes is a possible solution [20]. Examples of successful alternative living donation programmes are national kidney-exchange [21], ABO incompatible programmes [22], and domino-paired anonymous donation [23]. Involving patient groups in home-based education programmes is another possible solution. By delivering an educational program in the patient's home, it is possible to more effectively reach specific patient groups and their family and friends about the option of living donation [24].

## Figures and Tables

**Table 1:** Interview topics and example questions.

Topic	Example question
Demographic variables	-What education did you follow? -What is your current job?

Organ trade and trafficking in the Netherlands	-Do you know any cases involving illegal organ trade or trafficking in the Netherlands? -Can you describe these cases?

Transplant tourism: number and nature	-Are there patients who are removed from the waiting list (with the exception of those who died) who have not received a deceased or living donor organ? -Are there patients who have gone abroad for organ transplants? -Are there patients who have gone abroad to purchase an organ for transplant? -How do you know the organs are purchased? -How often do patients from your centre go abroad for paid organ transplants? -Are these cases registered? -How do these patients find information about transplants abroad?

Patient demographics	-What is the age, sex, and nationality of these patients?

Destination countries	-Which countries did these patients travel to?

Medical care	-Do these patients need medical care when they return? -What kind of complications occurred? -Do you provide medical care? -What does this care involve? -Do you help patients before they go abroad? -What kind of help do you give them? -What are your reasons to help them? -What are your reasons not to help them?

Information about the donor	-Is there information available about the donor in the returning patient's medical record? -If so, what kind of information?

Information about the hospital and physician abroad	-What kind of information is known about the hospital and/or physicians abroad?

**Table 2:** Definition and prohibition of transplant tourism.

World Health Assembly Resolution 57.18—Human organ and tissue transplantation
“The Fifty-seventh World Health Assembly […] urges Member States […] to take measures to protect the poorest and vulnerable groups from “*transplant tourism*” and the sale of tissues and organs, including attention to the wider problem of international trafficking in human tissues and organs”;



The Declaration of Istanbul on Organ Trafficking and Transplant Tourism

*Definition*
**“** *Travel for transplantation* is the movement of organs, donors, recipients or transplant professionals across jurisdictional borders for transplantation purposes. Travel for transplantation becomes *transplant tourism* if it involves organ trafficking and/or transplant commercialism or if the resources (organs, professionals and transplant centers) devoted to providing transplants to patients from outside a country undermine the country's ability to provide transplant services for its own population.”



*Principle 6*
“Organ trafficking and transplant tourism violate the principles of equity, justice and respect for human dignity and should be prohibited.”
